# Neural tube defect among newborns in public hospitals of Tigray region, northern Ethiopia: A cross-sectional study

**DOI:** 10.1371/journal.pone.0330937

**Published:** 2025-09-17

**Authors:** Goitom Girmay, Berhane Hailu Gebrezgi, Hiwot Gebrewahid, Aman Teklu, Guesh Mebrahtom, Gebrekiros Gebremichael Meles, Ebud Ayele Dagnazgi, Fireweyni Fisshatsion, Birhane Mekonen Negash

**Affiliations:** 1 Department of Midwifery, College of Health Sciences, Aksum University, Aksum, Ethiopia; 2 Department of Adult Health, School of Nursing, Aksum University, Aksum, Ethiopia; 3 School of Public Health, College of Health Sciences, Mekelle University, Mekelle, Ethiopia; 4 Department of Public Health Nutrition, College of Health Sciences, Aksum University, Aksum, Ethiopia; 5 School of Medicine, College of Health Sciences, Aksum University, Aksum, Ethiopia; University of Oulu: Oulun Yliopisto, FINLAND

## Abstract

**Background:**

Neural tube defects are serious congenital abnormalities caused by abnormal neural tube closure that occur between third and fourth weeks of pregnancy. Globally, neural tube defect is one of the top causes of morbidity and mortality among children under the age of five years. Ethiopia bears the highest adjusted mortality rate attributable to neural tube defects among sub-Saharan African countries. Despite this burden, neural tube defects remain understudied in Tigray, a region recently devastated by conflict.

**Objective:**

To assess the prevalence and factors associated with neural tube defect among newborns in public hospitals of Tigray, northern Ethiopia, 2024

**Methods:**

A facility based cross sectional study design was conducted among 1155 newborns in randomly selected public hospitals in the Tigray region from April 1 to May 30, 2024. Study participants were selected using a systematic random sampling technique. Data were collected through interviewer-administered, pretested, and structured questionnaires. Variables with a p-value of less than 0.25 in the bivariate logistic regression were included in the multivariable analysis to assess their independent effects. Associations between dependent and independent variables were considered statistically significant at a p-value < 0.05.

**Results:**

The overall prevalence of neural tube defects in this study was 3%. Residence (AOR=3.37, 95% CI: 1.46-7.77), folic acid supplementation before and during pregnancy (AOR=0.14, 95% CI: 0.06-0.33), having no medical illness during pregnancy (AOR=0.10, 95% CI: 0.04-0.21), food consumption score (AOR=2.90, 95% CI: 1.10-7.82), and alcohol consumption during pregnancy (AOR=2.90, 95% CI: 1.30-6.45) were the determinants for neural tube defects.

**Conclusions:**

The prevalence of neural tube defects is comparatively high among newborns born in Tigray’s public hospitals as compared to previous studies. Residing in rural areas, folic acid supplementation before and during pregnancy, having no medical illnesses during pregnancy, poor food consumption scores, and alcohol consumption during pregnancy were the determinants for neural tube defects. Preventive strategies, such as periconceptional folic acid supplementation, folate fortification, promoting healthy dietary practices, avoiding alcohol consumption, early maternal screening, and treatment of medical illnesses, are essential at both regional and national levels.

## Introduction

Neural tube defects (NTDs) are congenital anomalies that result from early malformation in the development of the brain and spinal cord [1]. NTDs occur during the third and fourth weeks of pregnancy [2]. NTDs are classified into two categories: closed NTDs, where tissue covers the exposed neural tissue, and open NTDs, where the neural tissue remains visibly uncovered [3]. The three primary subtypes of NTDs include anencephaly, encephalocele, and spina bifida [4]. NTDs are a major contributor to global under-five mortality and morbidity with an estimated prevalence of 18.6 cases per 10,000 live births [5]. Annually, theses defects impact 300,000 newborns worldwide, accounting for 88,000 deaths and 8.6 million disability-adjusted life years [6]. Regional disparities are stark: in Africa, NTD prevalence ranges from 9.80 to 50.71 per 10,000 live births, five times higher than in Western nations with folic acid supplementation programs [1,7–9]. Ethiopia faces NTD burden, with reported prevalence rates ranging from 63.3 to 571 cases per 10,000 live births [3,10–13]. Surveillance data from sub-Saharan Africa and South-east Asia highlight Ethiopia’s disproportionate mortality rates: 7.5% of under-five deaths and 104 NTD-attributable deaths per 10,000 live births; the highest in these regions [14]. Pre-war Tigray exemplified this crisis, with NTD prevalence at 1.31–2.15% (131–215 per 10,000), 26–43 times higher than the global target of 5 cases per 10,000 achievable through folic acid fortification [12,13]. Folate deficiency remains a critical risk factor for NTDs in Ethiopia, with 20.8% of women of reproductive age exhibiting inadequate folate levels [15]. Compounding this issue, only 1.92% of women adhered to folic acid supplementation during the recommended periconceptional period; a key preventive measure, highlighting a dire gap in prenatal care [16].

NTDs extend beyond physical health outcomes, imposing profound emotional distress, psychological trauma, and financial strain on affected families. These lifelong disabilities correlate not only with elevated mortality and morbidity but also with dropping socioeconomic challenges, including caregiver burnout, lost income, and stigmatization [17,18]. Research has identified multiple modifiable and non-modifiable risk factors for NTDs during pregnancy, including; folic acid deficiency (the most significant preventable cause), substance abuse (alcohol/drugs), inadequate antenatal care, advanced maternal age, history of stillbirths, environmental exposures (e.g., radiation, pesticides), and excessive coffee consumption [1,8,19].

Despite the Ethiopian governments efforts to ease NTDs through public health strategies, including mandatory food fortification, double fortification of salt, and nutrition education and awareness [20], these initiatives have been critically undermined by the war broke out in 2020 in Tigray region. The conflict displaced healthcare workers, collapsed medical infrastructure, and triggered a prolonged blockade that severed access to folate-rich foods, prenatal supplements, and antenatal care services essential for NTD prevention [21].

The post-war toll on NTDs in Tigray remains unstudied, as no studies have investigated the region since the war. The near-total collapse of healthcare infrastructure, coupled with a two-year blockade that halted access to folate-fortified foods, prenatal supplements, and maternal care, has likely exacerbated modifiable risk factors such as malnutrition and antenatal care gaps. This humanitarian crisis underscores an urgent need to address preventable birth defects in a population already disproportionately burdened by pre-war NTD rates. Therefore, this study aims to assess the magnitude and factors associated with NTDs among newborns in public hospitals across the Tigray region.

## Materials and methods

### Study area and period

This study was conducted in public hospitals across Tigray, a region in northern Ethiopia located approximately 780 kilometers from Addis Ababa, the nation’s capital. Prior to the 2020–2022 war, Tigray had a population of 9.4 million and a total fertility rate of 4.6 children per woman [21]. The region’s healthcare infrastructure included 14 hospitals, 170 health centers, and 552 health posts, forming a network critical for maternal and neonatal care. In November 2020, escalating tensions between the Tigray regional government and the Ethiopian federal government; supported by Eritrean and Amhara forces; erupted into full-scale war. The conflict resulted in over 600,000 fatalities and catastrophic damage to Tigray’s healthcare system: 89% of health facilities were looted, destroyed, or rendered nonfunctional by allied forces [21].

The study was conducted from April 1/2024 to May 30/2024.

### Study design

Facility based cross sectional study design was applied.

### Source population

All pregnancies reaching ≥12 weeks gestation; whether terminated or delivered in Tigray’s public hospitals during the study period were included.

### Study population

All selected pregnancies reaching ≥12 weeks gestation; whether terminated or delivered in Tigray’s public hospitals during the study period were included.

### Eligibility criteria

#### Inclusion criteria.

All pregnancies terminated or were delivered in public hospitals of the Tigray region after 12 weeks with their mothers during the data collection period.

#### Exclusion criteria.

The study excluded newborns whose mothers were unavailable or unable to be interviewed due to illness.

### Sample size determination

The required sample size for this study was determined using single population proportion formula.


$$𝐧=\frac{(z{\frac{\propto }{2})}^{2}p\left(1-p\right)}{d^{2}}$$

**where;**

n = minimum required sample size

Z α/2 = standard score corresponding to 95% CI,

P = is the proportion of NTDs from a previous study done in the Tigray region, northern Ethiopia (2.15%) [12].

d = is the margin of error. Since P is < 10%, “d” was calculated as d = P/2, d = 0.0215/2 = 0.01075


$$\text{n}=\frac{\left(1.96{)}^{2}*0.0215(0.9785\right)}{0.01075^{2}}=700$$

Considering a 10% non-response rate added, 770 is the final sample size.

1155 was the final sample size after taking into account the design effect of 1.5.

### Sampling technique and procedure

**Fig 1:**
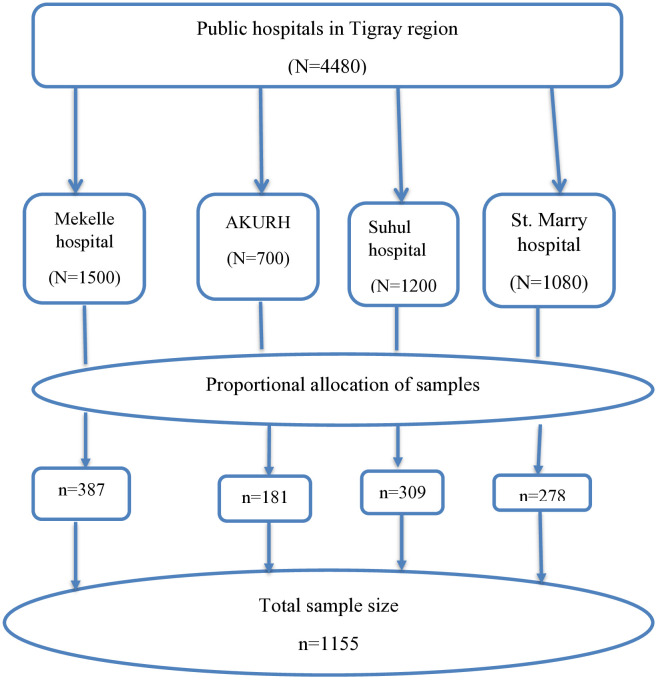
Schematic presentation of sampling procedure to assess magnitude and associated factors of NTDs in public hospitals of the Tigray region, Ethiopia, 2024.

From all hospitals operating under the interim government of the Tigray region, four hospitals (Mekelle Hospital, Aksum University Referral Hospital, Suhul Hospital, and St. Mary Hospital) were selected using a simple random sampling method. Each selected hospital was allocated a proportionate share of the sample size: Mekelle Hospital = 387, Aksum University Referral Hospital = 181, Suhul Hospital = 309 and St. Mary Hospital = 278. A systematic random sampling method was employed to select the study participants. In the selected hospitals, the average monthly birth rate from the previous year was determined. The sampling interval (K^th^) was calculated by dividing the two-month birth rate (N) by the total sample size (n), using the formula: K^th^ = N/n, where N = 4,480 and n = 1,155. Thus, K^th^ = 4,480/1,155 ≈ 4. This means every fourth newborn was included in the study. The random starting point was selected using the lottery method (Fig 1).

### Operational definitions

**Neural tube defect:** Classified as Present/Absent. The presence or absence of neural tube defect was confirmed by the physician in duty. The presence of NTDs comprises at least any one of anencephaly or spina bifida or encephalocele [17].

**Food Consumption Score (FCS):** This is a score calculated based on the frequency of consumption of various food groups by a household during the seven days preceding the survey. After calculating the FCS, specific thresholds are established to categorize the results

0-21=Poor, 21.5-35=Borderline, > 35=Acceptable [22]

**Low birth weight**: Low birth weight neonates were sub grouped according to the degree of smallness at first weight determination after birth.

✓ Low birth weight < 2500 gram, ✓ Very low birth weight < 1500 gram, ✓extremely low birth weight < 1000 gram

**Preconception care:** It is a set of interventions that are to be provided before pregnancy, to promote the health and well-being of women and couples, as well as to improve the pregnancy and child-health outcomes [23].

### Data collection tool and procedure

The questionnaire used was developed from different literatures conducted in Ethiopian and other African countries [3,4,6,17,24,25]. Initially created in English, it underwent translation into the local language, Tigrigna, by qualified language experts. To ensure accuracy and consistency, these experts then translated back the Tigrigna version into English. Data collection employed structured, interviewer-administered questionnaires that were pretested prior to the main study. Additionally, chart reviews were used to obtain specific maternal and newborn variables. Dietary intake during pregnancy was assessed using a FCS chart, adapted from the World Food Programme [22]. The study employed three supervisors and four data collectors, all of whom held Bachelor of Science (BSC) degrees in midwifery, to carry out data collection in the study areas were trained on each item included in the questionnaires and get consent from the study participants and collect data after the physician had completed the history and physical examination.

### Data quality control

A pretest was conducted on 5% of the total sample size, involving 58 participants, at Adwa Hospital two weeks prior to the actual data collection period. To ensure linguistic accuracy and conceptual equivalence, the questionnaires were initially prepared in English, translated into Tigrigna by language experts, and then back-translated into English to verify consistency with the original version. Data collectors and supervisors underwent a full-day training session, which covered interviewing techniques, the study’s objectives, and the different sections of the questionnaire. The principal investigator reviewed each questionnaire both during and after data collection to ensure completeness and maintain data quality.

### Data management and analysis

The filled questionnaires were checked for completeness, and entered in to EpiData manager version 4.4.2.1 and analyzed using SPSS version 29 statistical package. Binary logistic regression with a 95% confidence interval (CI) was used to examine the relationship between the outcome and explanatory variables. Initially, bivariate logistic regression was performed, and variables with a P-value ≤ 0.25 were included in the multivariable analysis to assess their independent effects. Multicollinearity was evaluated using the variance inflation factor (VIF), and the Hosmer-Lemeshow test was employed to assess the model’s goodness-of-fit. Variables with a P-value < 0.05 were considered statistically significant in determining the association between the dependent and independent variables. Finally, texts, frequency tables, and mean were used to report the result.

### Ethical considerations

This study received ethical approval from the Institutional Review Board (IRB) of Aksum University College of Health Sciences (Ref. No: 035/2024) on 29/02/2024. Formal permission was secured from administrative authorities at all participating hospitals prior to data collection. Written informed consent was obtained from all participants, including guardians of newborns, after explaining the study’s purpose, procedures, risks, and benefits. Participation was voluntary, and respondents retained the right to withdraw at any stage without consequence. To ensure confidentiality, all data were anonymized at the point of collection, with participant identifiers replaced by coded numbers. For illiterate participants, consent was obtained via thumbprint in the presence of an impartial witness.

## Results

### Socio-demographic characteristics

A total of 1155 mothers were enrolled in the study, which made response rate of 100%. The mean age of the mothers was ± SD age of the mothers 28 years (± 5.4). Regarding religious affiliation, 1,121 (97%) participants were Orthodox Christians. Of the mothers involved in the study, 969 (83.6%) resided in urban areas. More than half of the newborns, 651 (56.4%), were male (Table 1).

**Table 1:** Socio-demographic characteristics of study participants in public hospitals of the Tigray region, Ethiopia, 2024.

Variable	Category	Frequency	Percent (%)
Maternal age	<20	103	8.9
	21–25	284	24.6
	26–30	471	40.8
	31–35	171	14.8
	>35	126	10.9
Religion	Orthodox	1121	97.0
	Muslim	33	2.9
	Protestant	1	0.1
Residence	Rural	189	16.4
	Urban	966	83.6
Maternal educational status	No formal education	63	5.5
	Elementary school	334	28.8
	High school	442	38.3
	College and above	316	27.4
Occupation	Government employee	117	10.1
	Private employee	60	5.2
	Merchant	169	14.6
	House wife	772	66.8
	Student	32	2.8
	Jobless	5	0.4
Marital relationship	In marital relationship	1103	95.5
	Not in marital relationship	52	4.5
Maternal age at first childbirth	15–24	767	66.4
	25–35	388	33.6
Sex of newborn	Male	651	56.4
	Female	504	43.6

### Maternal obstetric, medical and newborn related characteristics

In this study, the majority of mothers, 1,019 (89.1%), delivered at term pregnancy. Of the mothers, 1,121 (97.1%), attended ANC follow-up. Nearly all mothers, 1,134 (98.2%), reported no preconception care. A significant proportion of mothers, 1,044 (90.4%), were supplemented with folic acid or multivitamins before and during pregnancy. Only 125 (10.8%) mothers experienced medical illness during pregnancy (Table 2).

**Table 2:** Maternal obstetric, medical and newborn related characteristics of study participants in public hospitals of the Tigray region, Ethiopia, 2024.

Variable	Category	Frequency	Percent (%)
Gestational age in weeks	<34	64	5.5
	34–36	62	5.4
	37–42	1029	89.1
Parity	1–2	748	64.8
	3–4	325	28.1
	> 4	82	7.1
Antenatal care follow-up for the index pregnancy	Yes	1121	97.1
	No	34	2.9
Planned pregnancy	Yes	889	77.0
	No	266	23.0
Preconception care	Yes	21	1.8
	No	1134	98.2
Folic acid supplemented prior to and during pregnancy	Yes	1044	90.4
	No	111	9.6
Previous history of NTD	Yes	4	0.3
	No	1151	99.7
Family history of NTD	Yes	1	0.1
	No	1154	99.9
History of stillbirth	Yes	36	3.1
	No	1119	96.9
History of abortion	Yes	158	13.7
	No	997	86.3
The duration of each child’s previous breastfeeding	No previous child	332	28.7
	≤2 years	770	66.7
	>2 years	53	4.6
Birth weight	1–1.499	6	0.5
	1.5–2.499	104	9.0
	2.5-5	1045	90.5
Medical illness during pregnancy (Anemia, APH, Asthma, CHF, DM, GDM, Malaria, Hepatitis B infection, HTN, PIH, PID, PUD, STI and UTI)	Yes	125	10.8
	No	1030	89.2

**Key;** APH: Antepartum hemorrhage, CHF: Congestive heart failure, DM: Diabetes mellitus, GDM: Gestational diabetes mellitus, HTN: Hypertension, PID: Pelvic inflammatory disease, PUD: Peptic ulcer disease, STI: Sexual transmitted disease, UTI: Urinary tract infection

### Maternal life style, behavioral and nutritional characteristics

Only 368 (31.9%) mothers had an acceptable food consumption scores. In this study, 223 (19.3%) mothers reported consuming alcohol at some point during their index pregnancies. Nearly half of the study participants, 525 (45.5%) stated that they consumed three or more cups of coffee daily (Table 3).

**Table 3:** Maternal lifestyle, behavioral, and nutritional characteristics of study participants in public hospitals of the Tigray region, Ethiopia, 2024.

Variable	Category	Frequency	Percent (%)
Food consumption score	Poor	414	35.8
	Border line	373	32.3
	Acceptable	368	31.9
Alcohol consumption	Yes	223	19.3
	No	932	80.7
Daily coffee consumption	No regular daily consumption	519	44.9
	1–2 cups per day	111	9.6
	≥3 cups per day	525	45.5
Preconception tea use	Yes	246	21.3
	No	909	78.7
Exposure to radiation	Yes	11	1
	No	1144	99
Exposure to pesticides	Yes	27	2.3
	No	1128	97.7
Smoking during pregnancy	Yes	11	1
	No	1144	99
Passive exposure to cigarette	Yes	26	2.3
	No	1129	97.7

### Prevalence of neural tube defects in public hospitals of Tigray region, Ethiopia

Among the 1,155 newborns and terminated pregnancies included in the study, 35 cases of NTDs were identified, yielding an overall prevalence of 3% (95% CI: 2.5–4.2%). Among the 1,155 participants, 1,120 (97.0%) had no NTDs. Of the 35 identified NTD cases: 16 (1.4%) were anencephaly, 14 (1.2%) spina bifida, and 5 (0.4%) encephalocele.

The prevalence of anencephaly was 1.4% (95% CI: 0.8%–2.2%), while spina bifida had a prevalence of 1.2% (95% CI: 0.7%–2.0%), and encephalocele accounted for 0.4% (95% CI: 0.1% –1.0%).

**Fig 2:**
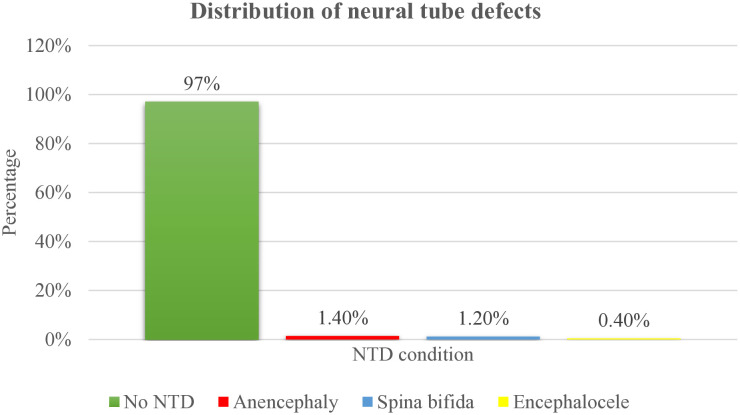
NTD distribution among the study participants in public hospitals of Tigray region, Ethiopia, 2024.

Among the 35 identified cases of NTDs, 26(74.3%) resulted in medical termination of pregnancy following antenatal diagnosis. The remaining 9(25.7%) progressed to term delivery without prior antenatal NTD diagnosis; of these, 3 (33.3%) were stillbirths (Fig 2).

### Factors associated with NTDs in public hospitals of Tigray region, Ethiopia

In the bivariable logistic regression analysis, variables such as residence, maternal educational status, newborn sex, pregnancy planning, folic acid supplementation before and/or during pregnancy, medical illness during pregnancy, alcohol consumption during pregnancy, and food consumption score showed a significant association with NTDs at a p-value of ≤0.25. As a result, all these variables were included in the multivariable logistic regression analysis. The multivariable analysis revealed that residence (AOR = 3.37, 95% CI: 1.46–7.77), folic acid supplementation before and/or during pregnancy (AOR = 0.14, 95% CI: 0.06–0.33), medical illness during pregnancy (AOR = 0.10, 95% CI: 0.04–0.21), food consumption score (AOR = 2.90, 95% CI: 1.10–7.82), and alcohol consumption during pregnancy (AOR = 2.90, 95% CI: 1.30–6.45) were significantly associated with NTDs (Table 4)

**Table 4:** Bivariable and multivariable logistic regression analysis of factors associated with NTDs in public hospitals of the Tigray region, Ethiopia, 2024.

Variable	Category	NTD		COR	AOR	P-value
		Yes	No			
Residence	Rural	17(48.6%)	172(15.4%)	5.2(2.6–10.3)	3.37(1.46–7.77)	**0.004**
	Urban	18(51.4%)	948(84.6%)	1	1	
Maternal educational status	No formal education	08(22.9%)	55(4.9%)	7.5(2.5–22.5)	2.50(0.57–9.53)	0.194
	Elementary school	10(28.6%)	324(28.9%)	1.6(0.6–4.4)	0.82(0.26–2.55)	0.735
	High school	11(31.4%)	431(38.5%)	1.3(0.5–3.6)	0.96(0.32–2.88)	0.964
	College and above	06(17.1%)	310(27.7%)	1	1	
Sex of newborn	Male	23(65.7%)	628(56.1%)	1	1	
	Female	12(34.3%)	492(43.9%)	0.7(0.3–1.4)	0.63(0.29–1.40)	0.253
ANC follow up	Yes	31(88.6%)	1090(97.3%)	1	1	
	No	04(11.4%)	30(2.7%)	4.7(1.6–14.1)	0.99(0.24–4.22)	0.995
Planned pregnancy	Yes	23(65.7%)	866(77.3%)	1	1	
	No	12(34.3%)	254(22.7%)	1.8(0.9–3.6)	1.45(0.65–3.23)	0.364
Folic acid supplemented prior and during pregnancy	Yes	19(54.3%)	1025(91.5%)	0.1(0.06–0.22)	0.14(0.06–0.33)	**<0.001**
	No	16(45.7%)	95(8.5%)	1	1	
Medical illness during pregnancy	Yes	16(66.7%)	109(9.7%)	1	1	
	No	19(33.3%)	1011(90.3%	0.13(0.06–0.3)	0.10(0.04–0.21)	**<0.001**
Food consumption score	Poor	20(57.1%)	394(35.2%)	2.62(1.1–6.3)	2.90(1.10–7.82)	**0.035**
	Border line	08(22.9%)	365(32.6%)	1.13(0.4–3.2)	1.64(0.52–5.14)	0.396
	Acceptable	07(20.0%)	361(32.2%)	1	1	
Alcohol consumption	Yes	14(40.0%)	209(18.7%)	2.60(1.13–6.06)	2.90(1.3–6.40)	**0.009**
	No	21(60.0%)	911(81.3%)	1	1	

Key: ANC: Antenatal care, COR: Crude odds ratio, AOR: Adjusted odds ratio.

## Discussion

This study assessed the prevalence and factors associated with NTDs. This study indicated that the prevalence of NTDs in public hospitals of the Tigray region was 3% (95% CI: 2.5%−4.2%). Residence, folic acid supplementation, medical illnesses during pregnancy, food consumption score and alcohol consumption during pregnancy were the factors associated with NTDs in public hospitals of Tigray region.

The prevalence of NTDs in this study was significantly higher than findings from prior studies conducted in Tigray hospitals (2.15%) [12], Debre Berhan referral hospital (1.09%) [26], Addis Ababa (1.66%) [27], public referral hospitals in eastern Ethiopia (0.2%) [6], Hargeisa, Somaliland (0.16%) [28], Kampala, Uganda (0.10%) [29], Europe (0.09%) [30], Nicaragua (0.14%) [31], and several meta-analyses conducted in Ethiopia, and Africa which range from 0.21% to 0.83% [1,7,8,10,32,33]. This elevated prevalence may reflect the socioeconomic and nutritional crises exacerbated by the recent war in the Tigray region, which likely disrupted healthcare access, food security, and maternal health services. However the result of this study is lower than the study done at Hiwot Fana specialised University Hospital, Harar, Ethiopia (5.71%) [3]. This discrepancy may stem from differences in study populations: the study conducted at Hiwot Fana specialised University Hospital focused on neonates admitted to a neonatal intensive care unit, which typically represents a higher-risk cohort with severe congenital anomalies. Regarding NTD sub-types, the prevalence of anencephaly in this study was 1.4%. This is higher than the rates reported in studies conducted at Debre Berhan Referral Hospital, North Shewa, Ethiopia (0.56%) [26], Nicaragua (0.05%) [31], and multiple meta-analysis studies across Ethiopia and Africa which ranged from 0.09% to 0.23% [7,10,32]. In this study, the prevalence of spina bifida was 1.2%. This finding is higher than those reported in studies conducted at Debre Berhan Referral Hospital, North Shewa, Ethiopia (0.35%) [26], Hargeisa, Somaliland (0.03%) [28], Nicaragua (0.08%) [31], and meta-analysis studies in Ethiopia and Africa, which reported prevalence rates ranging from 0.17% to 0.41% [7,8,10,32]. Additionally, the prevalence of encephalocele in this study was 0.4%, which is higher than the studies done in Debre Berhan Referral Hospital, North Shewa, Ethiopia (0.11%) [26], Nicaragua (0.73%) [31] and various meta-analysis in Ethiopia and Africa, which are in the range of 0.01% to 0.04% [7,8,10,32]. The observed differences in the prevalence of NTD subtypes may be due to the socio-economic and nutritional crisis precipitated by the two-year conflict in the Tigray region. The war has severely disrupted healthcare infrastructure, limited access to prenatal care and folic acid supplementation, and exacerbated food insecurity; all critical determinants of maternal and fetal health [34].

This study found that mothers residing in rural areas had threefold higher odds of delivering newborns with NTDs compared to their urban counterparts. This finding aligns with studies conducted in the Amhara [17] and Tigray [24] regions of Ethiopia, as well as nationwide systematic review and meta-analysis in Ethiopia [32]. The disparity may be due to limited access to healthcare services, nutritional deficiencies, and lower awareness of preventive measures in rural communities. These challenges are likely to be worsened by the two year war in Tigray region, which has severely disrupted healthcare infrastructure [35].

In this study, women who were supplemented with folic acid before and/or during pregnancy had an 86% lower risk of having newborns with NTDs than those who did not supplement. This finding is consistent with studies conducted in the Amhara region [17], Debre Berhan specialised hospital [4], and north Shoa zone hospitals [25], as well as meta-analysis studies in Ethiopia and across Africa [1,8–10,32]. Scientific evidence supports that folic acid plays a critical role in nucleotide synthesis, facilitating neural tube closure and central nervous system development. Additionally, folate enhances acute signaling in neurons [9].

This study found that women who did not experience medical illnesses during pregnancy were 90% less odds of delivering newborns with NTDs compared to those who did. This finding is supported by studies conducted in the Amhara region [17] and a meta-analysis [32] in Ethiopia. Medical illnesses such as diabetes mellitus during pregnancy can disrupt normal cell division in the developing neural tube, leading to improper closure and the formation of NTDs(32). Furthermore, pregnant women with medical conditions may experience reduced food intake due to loss of appetite or other factors, potentially lowering their folic acid levels and exacerbating the risk of NTDs.

In this study, women with poor food consumption during pregnancy were three times more likely to have newborns with NTDs compared to women who had acceptable food consumption. The result is consistent with the study conducted in eastern Ethiopian public referral hospitals [6]. The two-year civil war in the Tigray region has led to widespread food insecurity, affecting the entire population. The conflict has caused reduced agricultural production, market instability, and, mass displacement, resulting in severe hunger [36]. Consequently, undernourished pregnant women may have a decreased folic acid level in their bodies, which can lead to NTDs in their newborns.

This study identified a significant association between alcohol consumption during pregnancy and NTDs. Mothers who consumed alcohol had threefold higher odds of delivering a newborn with NTDs compared to abstainers. This finding is consistent with meta-analyses in Ethiopia and across Africa [8,32]. The association may be explained by the fact that excessive alcohol consumption during pregnancy interferes with folic acid transport. Additionally, alcohol can induce NTDs by reducing retinoic acid signaling and promoting the expansion of the neural plate [32,37].

## Conclusions

The study highlights that, NTD is a significant health problem among newborns born in public hospitals of Tigray region, with several determinants identified. The prevalence of NTDs is comparatively high among newborns born in Tigray’s public hospitals as compared to previous studies. While living in rural areas, having poor food consumption scores, and drinking alcohol during pregnancy were risk factors for NTDs in newborns delivered in Tigray’s public hospitals, taking folic acid before and during pregnancy and not having any illnesses during pregnancy were found to be protective factors. Preventative strategies, such as periconceptional folic acid supplementation, folate fortification, and promoting healthy dietary practices, should be strengthened to reduce the burden of NTDs. Policies should be advocated to address disparities in healthcare services and to promote the importance of folic acid utilization, particularly in rural areas of the region. Implementing health education programs to raise awareness among pregnant women about avoiding consumption of alcohol, is essential to prevent adverse effects on fetal development and lifelong consequences for the child. Early maternal screening and treatment for medical conditions in women at higher risk of NTDs are potential strategies to reduce the burden of NTDs among women of reproductive age. To enhance the representativeness and generalizability of the study findings, further research involving a more diverse group of participants and a wider range of study areas is recommended.

### Strengths and limitations

#### Strengths.

The study’s outcome variable was based on physician diagnosis, ensuring accuracy and reliability in identifying cases of NTDs. This strengthens the validity of our findings. Our data collection process includes a wide range of socio-demographic, maternal obstetric, medical and newborn related and maternal lifestyle, behavioral and nutritional characteristics, allowing for a holistic view of the determinants of NTD. Note the 100% response rate, which enhances the representativeness of our sample and reduces selection bias. This study highlights Tigray’s postwar NTDs crisis using recent data

#### Limitations.

Since this study is facility-based, newborns delivered outside of health facilities were not included. As a result, the magnitude of NTDs may be misestimated. Maternal self-reporting of behaviors (e.g., alcohol use, preconception care) could introduce recall bias.
